# Toward a Variable Stiffness Surgical Manipulator Based on Fiber Jamming Transition

**DOI:** 10.3389/frobt.2019.00012

**Published:** 2019-03-19

**Authors:** Margherita Brancadoro, Mariangela Manti, Fabrizio Grani, Selene Tognarelli, Arianna Menciassi, Matteo Cianchetti

**Affiliations:** The BioRobotics Institute, Scuola Superiore Sant'Anna, Pisa, Italy

**Keywords:** soft robotics, surgical manipulator, variable stiffness system, jamming transition, minimally invasive surgery

## Abstract

Soft robots have proved to represent a new frontier for the development of intelligent machines able to show new capabilities that can complement those currently performed by robots based on rigid materials. One of the main application areas where this shift is promising an impact is minimally invasive surgery. In previous works, the STFF-FLOP soft manipulator has been introduced as a new concept of using soft materials to develop endoscopic tools. In this paper, we present a novel kind of stiffening system based on fiber jamming transition that can be embedded in the manipulator to widen its applicability by increasing its stability and with the possibility to produce and transmit higher forces. The STIFF-FLOP original module has been re-designed in two new versions to incorporate the variable stiffness mechanism. The two designs have been evaluated in terms of dexterity and variable stiffness capability and, despite a general optimization rule did not clearly emerge, the study confirmed that fiber jamming transition can be considered an effective technological approach for obtaining variable stiffness in slender soft structures.

## Introduction

Robots today rely on a long tradition in the use of rigid materials for the most of their body. The use of rigid materials implies the possibility to use some basic simplifications, assumptions, and conventions that can support their design. This framework can lead to very advanced and complex machines, but most of the times the effectiveness of the robot is still heavily relying on the control performance. This traditional approach for making intelligent machines has been questioned when roboticists started to look at natural agents (e.g., humans, animals, and even plants) and their interaction with the environment (Laschi and Mazzolai, 2016). Observing the key role played by soft and flexible structures within the body to cope with the unstructured and unpredictable environments in everyday tasks, roboticists started to re-think the basic principle for designing, manufacturing and controlling robots. This paradigmatic revolution is now known as soft robotics (Rus and Tolley, 2015). In this new paradigm, softness, and flexibility have acquired a strategic role for developing versatile, dexterous, and intrinsically safe systems (Shen, 2016), but the real game changer that makes soft robotics effective is the variable stiffness capability. This ability allows for soft robots to maintain their own structural strength without losing the capability of reversibly transit between a stiff state and a compliant one for a better adaptation of the shape to unstructured environments.

The attention of researchers approaching the design and manufacturing of a soft robot is firstly devoted to the definition of a body that counts on three main characteristics: shape, arrangement, and material properties of the constituting elements that serve, wherever it is possible, both as passive (structural) and active elements (Zambrano et al., 2014). This vision implies a significant increase in bodyware complexity, but also a simplification on control algorithms: a rich behavior does not necessarily come from a complex control, but may be the result of the interaction between body, control and environment (Pfeifer and Bongard, 2006).

In this framework, the innovative actuation technologies, investigated and developed by soft roboticists, represent the ground for a new generation of soft robots with advanced abilities, such as elongation, squeezing, growing, self-healing, and variable stiffness (Laschi et al., 2016). The topic is still an open issue. Literature reports some reviews and tentative approaches to identify, design and combine soft robotics technologies for stiffness tuning (Manti et al., 2016; Sun et al., 2017; Wang et al., 2018).

Among the most corroborated semi-active technologies for stiffness tuning (Manti et al., 2016), the material jamming transition has been largely investigated because of its simplicity, versatility, reversibility and possibility to customize the system according to the target application. The integration of a jamming mechanism into a device involves the presence of an external soft, elastic membrane filled with solid discrete material. At atmospheric pressure, the system presents high softness and compliance given by the fact that filler can easily and freely move inside the soft membrane; upon the application of vacuum, the membrane collapses on the filler material, thus freezing the dynamics of the overall system. Consequently, the material is densely packed and the friction prevents every kind of relative displacement. As a result, the entire structure behaves like a rigid material (Liu and Nagel, 1998).

The working principle that stands behind the phenomenon is today reproduced and frequently exploited in soft robotic systems at the macroscale; on the other hand, the physical principle that occurs at the microscale is still under investigation (Behringer and Chakraborty, 2018). Despite the fact that the technology is very easy to use and the advantages are undeniable, there is a lack of information on the underlying physical principle involved in the jamming transition and, as a consequence, there are no suitable models and tools able to guide the design choice, thus preventing its exploitation at market level (Amend et al., 2016).

Jamming transition has been investigated, for the first time, with grains encapsulated in an elastic membrane that, upon the application of vacuum, transit from a compliant state to a rigid one. This semi-active technology is commonly used in combination with other active actuation technologies to allow selective stiffening or shape locking of bending states in anthropomorphic grippers (Wall et al., 2015) or in highly articulated manipulators (Follmer et al., 2012). They can be also exploited as a mean to introduce selective anisotropies in the material behavior thus enabling locomotion patterns, such as rolling (Steltz et al., 2009), or vibration (Kaufhold et al., 2012). A widespread use of the technology is confirmed by its application as haptic or tactile interfaces (Follmer et al., 2012; Stanley et al., 2013; Li et al., 2014). The phenomenon has been then extended to the use of laminar material inside an elastic membrane in order to obtain layer jamming (Kim et al., 2013; Ou et al., 2014; Narang et al., 2018).

Literature analysis demonstrates that research prototypes are only based on granular and layer jamming, while the possibility of exploiting jamming transition using fibers as filler material is completely neglected. A preliminary study of this configuration has been investigated, for the first time, by Brancadoro et al. (2018): here, a comparative approach has been proposed to experimentally assess the performances of the jamming transition induced on fibers. In the same work, a first discussion on the main parameters affecting the system behavior has been introduced: fiber material, dimension, cross section and shape. The present paper builds upon the main results achieved in that previous work and focuses on the integration of a variable stiffness system based on fiber jamming transition in the STIFF-FLOP soft manipulator developed by the same research group (Abidi et al., 2018), hereafter called “original” to avoid confusion. This manipulator is based on three-flexible fluidic chambers that can be inflated to obtain omnidirectional bending and elongation. The STIFF-FLOP manipulator has already proved to introduce significant advantages into minimally invasive surgical procedures and specifically it has been successfully used as an endoscope in a total mesorectal excision procedure that was performed in two human cadaver models (Arezzo et al., 2017). Nevertheless, the manipulator has limited applications as surgical tool because of its poor capability of force application. This is the reason why, in its current configuration, it is best suited for endoscopic tasks, where safe interaction with organs and delicate tissues and dexterity are the main important features (Abidi et al., 2018). An endoscopic tool is devoted to inspection within the human body, thus it mainly requires dexterity and intrinsic safety (softness) in case of interaction with soft tissues. On the other hand, a surgical tool requires the ability of an effective interaction with human organs/tissues (e.g., for cutting, moving, pushing) thus it needs to be sufficiently rigid or (as in our case) the ability of tuning its stiffness. It implies that, in the two-module STIFF-FLOP surgical manipulator, the activation of the stiffening system of the proximal module is used to provide stabilization to the distal module while this latter is interacting with the tissues.

Earlier versions of the manipulator could count on a variable stiffness system based on granular jamming transition (Ranzani et al., 2015). It was effective and suitable for the surgical environment in terms of safety, but the miniaturization process revealed that this technology becomes very ineffective when used in almost 2D or 1D structures. Grains better act with 3D volumes, layers work well in planar structures while the mono-dimensionality of fibers is appropriate for long, slender systems. Thus, jamming transition based on fibers presents the right features for introducing a remarkable variable stiffness capability in the original STIFF-FLOP manipulator. Moreover, the manipulator is already driven by fluidic actuation technologies, thus the additional components needed to drive jamming transitions are limited.

With this in mind, the main driver of this study is the integration of a variable stiffness system in the STIFF-FLOP soft manipulator. This system already has the flexibility and dexterity needed for a safe tool for medical application. Moreover, the system is able to reach remote areas from different points of view using the same access port. The integration of the semi-active technology extends the already available functionalities of the system making possible also surgical actions.

**Figure 1:**
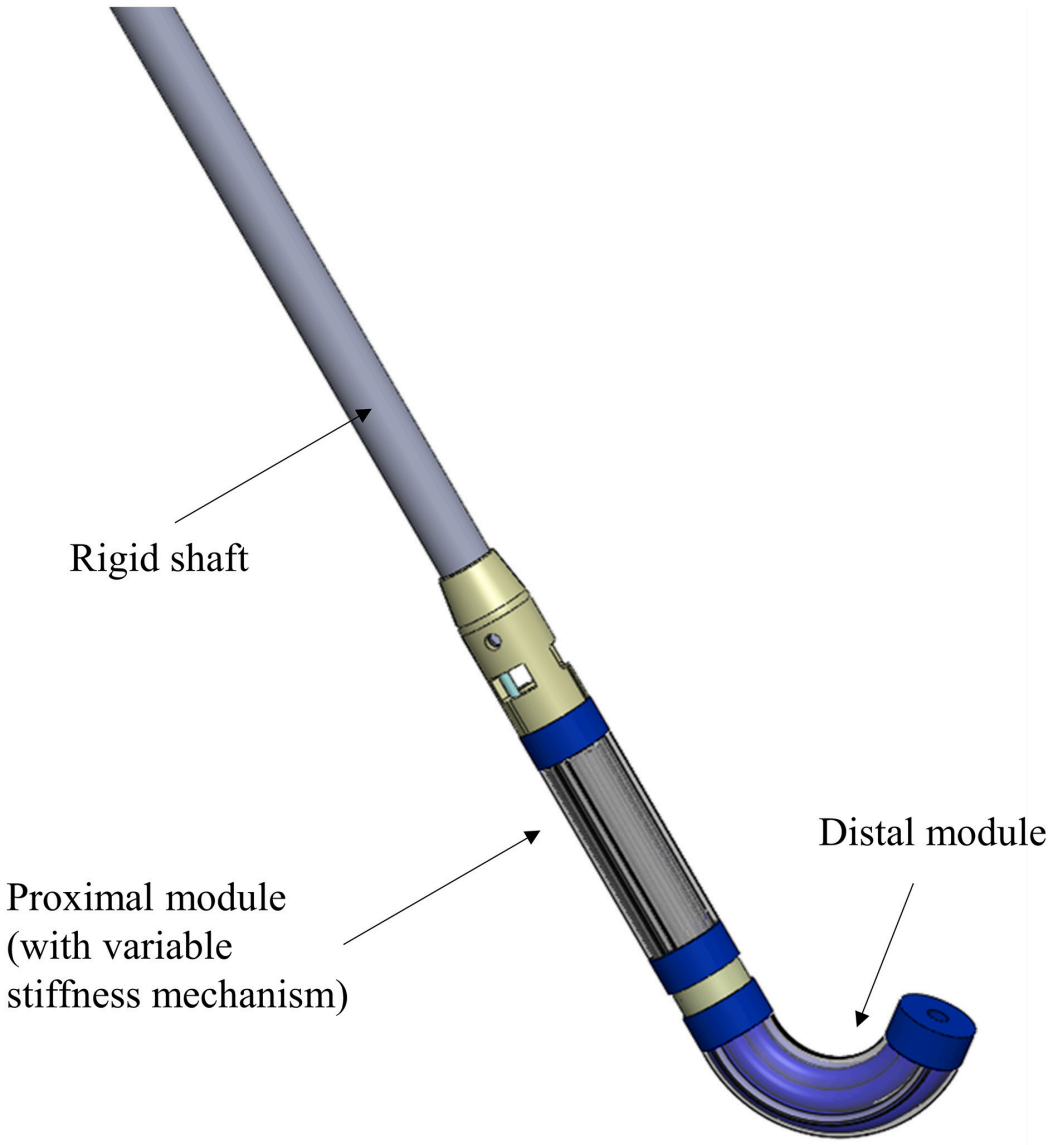
Representation of the two-module STIFF-FLOP manipulator attached to a rigid shaft used as support. The proximal module has been redesigned to lodge a variable stiffness system that can become rigid on demand and provide stability to the distal module (unvaried with respect to the original version).

In this framework, the new concept tested in the present work investigates the possibility to re-design the proximal module of the original STIFF-FLOP manipulator to integrate a variable stiffness system without affecting the original dimensions in terms of diameter and length. This will enable stiffness variation that provides support and acts as a stabilizer for the distal module, which in turn exploits its flexibility and dexterity to interact with organs or human tissues. The complete surgical manipulator proposed in Figure 1 will be composed of a proximal module (to be chosen between the two designs proposed in the present work) where the fiber jamming technology is integrated and a distal module that is the original one, as proposed in Abidi et al. (2018). The whole system could be then attached to a rigid shaft, which can be positioned and maneuvered at the insertion point by a surgeon or by a robot (Diodato et al., 2018) for performing minimally invasive surgery (MIS) procedures.

## Materials and Methods

Before presenting in detail the new design and the manufacturing procedure of the tested modules, it is worth briefly recapping the main characteristics the original STIFF-FLOP soft manipulator relies on. It includes three pairs of inflatable chambers, radially arranged around a central axis, and entirely made of silicone. These chambers are lined with a thin inextensible thread in a tight helical winding. The minimization of the helical pitch brings two advantages: it prevents a radial expansion and maximizes the longitudinal elongation when the chamber is under pressure (Fraś et al., 2015). This combination enables the possibility to obtain omnidirectional bending and longitudinal elongation depending on the pressure applied to each chamber. The chamber can be considered as an actuator that generates one motion primitive (MP). For continuum soft manipulators, the traditional degrees of freedom (DoFs) are replaced by MPs which result more appropriate for a soft robot that theoretically has infinite positions (Abidi et al., 2018). Considering this convention, the soft manipulator has three MPs for bending motion due to the inflation of each pair of chambers at different pressure values and one MP that describes the elongation due a simultaneous inflation of all the chambers at the same pressure value.

For this work, the original STIFF-FLOP module has been modified and declined into two different versions according to the following specifications:

- the dimensions of the module itself, in terms of external diameter and total length, remain constant in order both to pass through the trocar used in MIS and have comparable results with respect to the workspace covered by the original STIFF-FLOP module;
- the module should have at least one motion primitive to guarantee a minimum level of dexterity and flexibility;
- the module should have a free lumen.

The first one, referred as Module A in the next section, is based on the original design but hosts the fiber jamming system in the central channel (free lumen), not fulfilling the third requirement. The second one, referred as Module B, counts on a substantial revision of the actuation system: two pairs of fluidic actuators are substituted by two sites for fiber jamming, thus affecting the second requirement. While the omni directionality can be compensated externally (i.e., using the roll DoF of the rigid shaft reported in Figure 1), the free lumen is something that improves the system functionalities and surgeon's abilities.

Moreover, the integration of the variable stiffness system does not imply any modifications in terms of modularity of the overall final systems as described in Figure 1 or dimensions of the module itself. For this reason, the two designs here proposed as an alternative to the original proximal STIFF-FLOP module, are still compliant with the miniaturization constraints and, moreover, the covered workspace is comparable with the previous results.

The design and the functionalities of each module are described in the next two subsections while the manufacturing procedure, being mostly the same for both, is presented in a single subsequent section.

### Re-design of the Module

**Figure 2:**
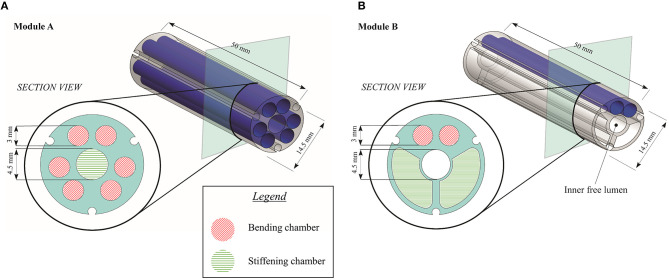
Design and section view of **(A)** Module A and **(B)** Module B.

***Module A***. The single module is 50 mm in length and 14.5 mm in external diameter thus resulting suitable for standard MIS applications (i.e., it is able to pass through a standard 15 mm trocar). Figure 2A shows the section view of the module containing three pairs of chambers, each measuring 3 mm in diameter and able to elongate only. With respect to the original version of the STIFF-FLOP module where the lumen (4.5 mm in diameter) was properly designed to allow the insertion of thin surgical equipment up to the tip, in the current version of the module the central channel hosts the fibers to produce the jamming transition (called “stiffening chamber”). With this design, the module is characterized by the variable stiffness functionality and by four MPs that supply the omnidirectional bending and the elongation.

***Module B***. The single module is again 50 mm in length and 14.5 mm in external diameter. With respect to Module A, this version has only one MP, supplied by a pair of chambers, while the remaining space is equally split into two chambers (each one with 27.41 mm^2^ in area) that host fibers for varying the module stiffness, as depicted in the section view reported in Figure 2B. This design implies that the module has one single bending plane and variable stiffness functionality, while the inner free lumen (4.5 mm in diameter) is preserved for inserting suitable surgical tools (e.g., graspers, mini ultrasound probes and radio-frequency tools), for housing electric wires (e.g., for a laparoscopic micro camera) or for routing pressure lines (in case of a multi-modules architecture).

### Fiber Selection

**Figure 3:**
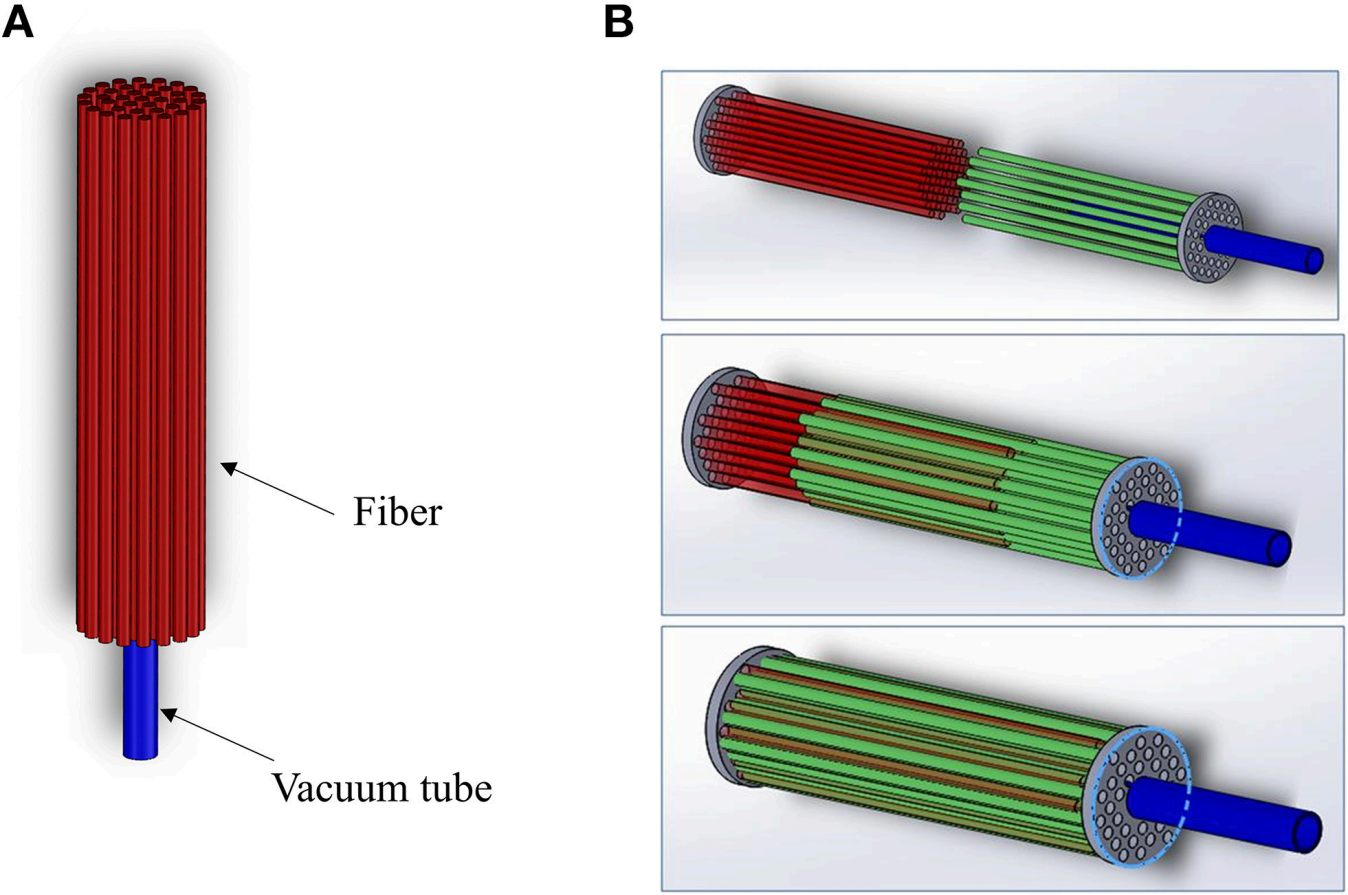
CAD model for the **(A)** BT and **(B)** CT joint.

The choice of the fibers to be used in the jamming-based system has been driven by the main achievements that authors have reported in the previous work (Brancadoro et al., 2018). In this earlier paper, a series of cylindrical samples made of a latex membrane filled with different fibrous materials has been tested and compared to identify the material that present the highest stiffness variation. In particular, PTFE, PVC, Nylon, Silicone, Waxed cotton, and Leather have been investigated and tested in two different configurations: bundle-type (BT) and comb-type (CT). The first one (i.e., BT) counts on fibers that are longitudinally arranged in a bundle fixed on one side only and without a specific organization (Figure 3A), while the CT configuration presents fibers organized as two tooth-interlocking combs (Figure 3B).

It is worth mentioning that, since the jamming transition is affected by friction effects among the filling fibers and between the fibers and the external membrane, the preliminary study proposed in Brancadoro et al. (2018) took this feature into account investigating the surface finishing of the selected fibers and its correlation with the jamming transition for the two configurations. In particular, despite a numerical analysis is still missing, in that paper all the materials selected for the preliminary assessment have been compared also from this point of view in order to relate the stiffness variation of the joint configuration (Bundle type BT or comb type CT) to the material type. In particular, authors noticed that the fiber roughness order has a role in the effectiveness of the jamming effect, being directly correlated with the sliding capability of the fibers. In the present study, authors decided to adopt the combination of joint configuration and fiber type that has demonstrated the best performances in terms of stiffness variation. Although other materials and configurations are possible, currently there is no specific study or general model, thus the choice can be only guided by a comparative experimental analysis. According to those results (summarized in Table 1), the waxed cotton in the CT configuration has demonstrated the most promising stiffening features (increase of 377.5%) and have been incorporated in the modules.

**Table 1:** Summary of the results from Brancadoro et al. (2018).

**Fiber material**	**Surface finishing**	**Design approach**	**Pressure (bar)**	**Force max. (N)**	**Stiffness variation *(F_**max jammed**_/F_**max unjammed**_)* (%)**
PTFE	Very smooth	BT	1.01325	5.33 ± 0.16	+93 ± 13
			0.1	10.26 ± 0.39	
		CT	1.01325	5.89 ± 0.7	+108 ± 27
			0.1	12.06 ± 0.12	
PVC	Smooth	BT	1.01325	2.75 ± 0.07	+180.5 ± 10.5
			0.1	7.70 ± 0.09	
		CT	1.01325	2.92 ± 0.08	+216.5 ± 14.5
			0.1	9.24 ± 0.17	
Nylon	Very smooth	BT	1.01325	8.01 ± 0.15	+135 ± 12
			0.1	18.79 ± 0.61	
		CT	1.01325	19.07 ± 1.59	+43 ± 19
			0.1	26.96 ± 1.32	
Silicone	Rough	BT	1.01325	4.25 ± 0.11	+89 ± 8
			0.1	8.01 ± 0.13	
		CT	1.01325	2.55 ± 0.35	+205 ± 44
			0.1	7.63 ± 0.05	
Waxed cotton	Very rough	BT	1.01325	2.65 ± 0.11	+254 ± 20
			0.1	9.36 ± 0.13	
		CT	1.01325	2.56 ± 0.02	+377.5 ± 7.5
			0.1	12.23 ± 0.10	
Leather	Very rough	BT	1.01325	3.54 ± 0.13	+79.5 ± 14.5
			0.1	6.35 ± 0.28	
		CT	1.01325	3.86 ± 0.07	+291.5 ± 26.5
			0.1	15.09 ± 0.76	

*The fiber type and the design approach selected for the present study is highlighted in the table (BT – Bundle Type; CT – Comb Type)*.

Fibers have been confined in the dedicated sites by using the same guidelines defined in the previous work in terms of packing factor (i.e., the volume of the fibers divided by the volume of the section). In particular, keeping the same packing factor, 8 fibers have been used for Module A while 14 fibers have been inserted into each chamber of Module B, since each fiber has a diameter of 0.9 mm.

### Manufacturing

**Figure 4:**
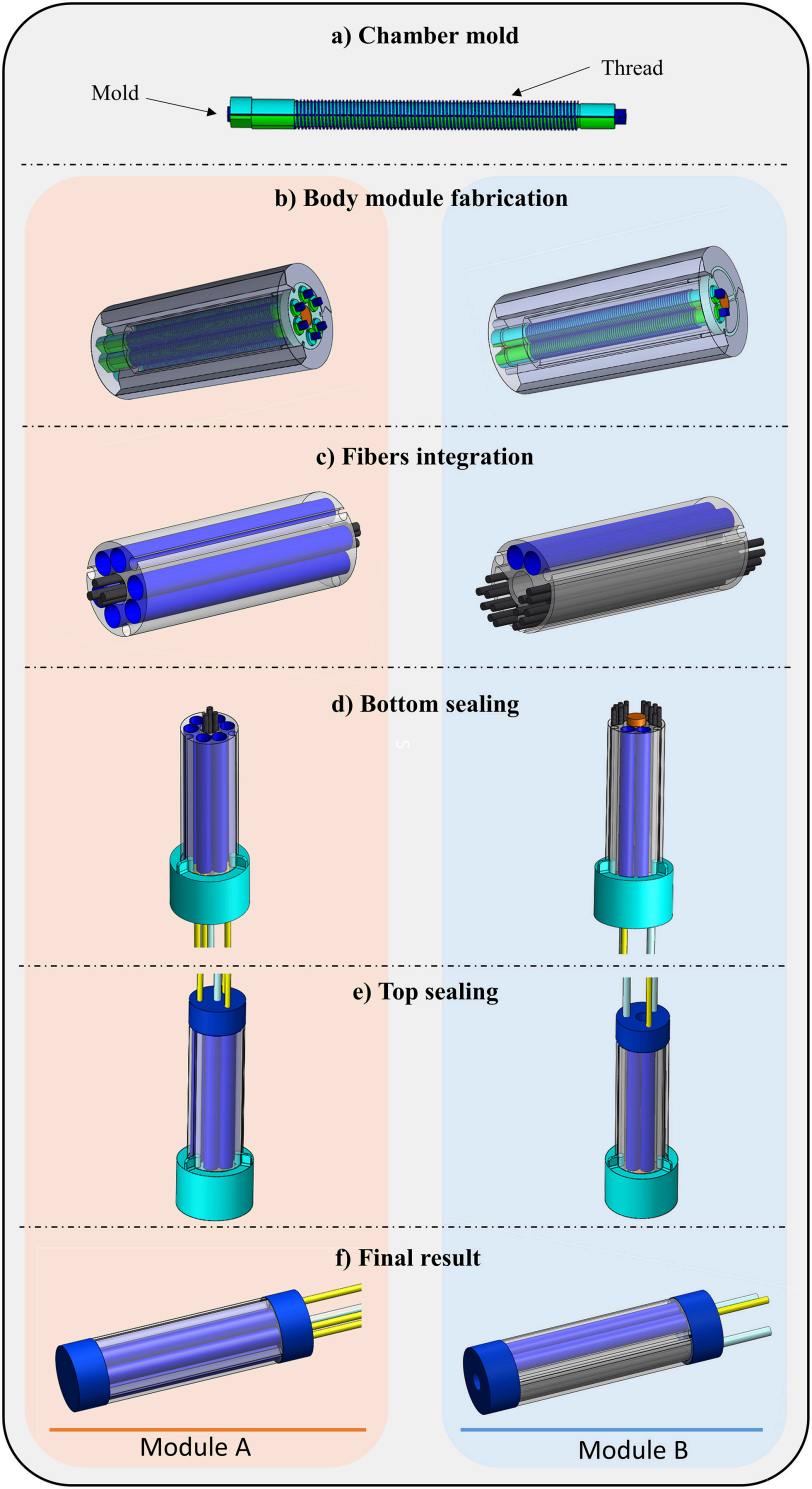
**(a–f)** Modules manufacturing steps.

The manufacturing of the two kinds of module consists of several steps based on silicone molding procedure. All the components for the module fabrication are realized using a 3D printer (ProJet MJP 3600, 3D Systems, South Carolina, US). For a better representation of the manufacturing procedure, the main phases are listed below and summarized in Figure 4:

- Firstly, the mold for the chambers is prepared by winding an inextensible polyester thread around a 3D printed cylinder. This cylinder is composed of three assembled parts, an inner core and two side parts (Figure 4a). Six chamber molds and two chamber molds are prepared for Module A and Module B, respectively.
- The fabrication of Module A starts positioning six chamber molds into a cylindrical-shaped mold composed of three identical parts and a central cylinder for the realization of the inner free lumen. To guarantee a precise mold alignment, that is essential for avoiding any asymmetries in the module, a thin Plexiglas plate is located on the top of the module for lodging all molds and for keeping them in place. The Plexiglas component is cut with a laser cutting machine (Universal Laser XLS10MWH, Universal Laser System Inc., US). The fabrication of Module B starts from the realization of the mold. It is composed of two chamber molds, two molds for the stiffening chambers, an inner cylinder and the upper Plexiglas plate for the alignment. Figure 4b shows the two assembly molds. Then, uncured silicone (Ecoflex 0050, Smooth On Inc., Macungie, PA) is poured into the molds and left to cure at room temperature. After the silicone has completely cured, all molds are removed.
- Once this step is completed, a total of 36 fibers (i.e., 8 fibers for Module A and 28 fibers for Module B) are inserted with a CT configuration in the lumen and in the two lateral chambers of the Module A and Module B, respectively (Figure 4c). For creating the CT configuration, in each module, half of fibers exceed of 3 mm from the bottom face of the module and the residual fibers exceed of the same length from the other side. In this way, it is possible to encapsulate the fibers into the silicone base, thus guaranteeing that the fibers are arranged as two tooth-interlocking combs.
- After fibers integration, the modules are sealed on bottom side using a dedicated cup mold filled with harder silicone (Smooth Sil 950, Smooth On Inc., Macungie, PA) (Figure 4d). At this stage, the pipes for the fluidic actuation (i.e., three for Module A and only one for Module B) and for the vacuum (i.e., one for Module A and two for Module B) are incorporated into the soft structure.
- The last step concerns the sealing of the top side of the modules and it follows the same procedure described above (Figure 4e). The fluidic chambers are connected in pairs through a small silicone pipe located internally as a bridge between chambers. Figure 4f shows the final result.

The weight of each module is 9.2 g and 7.6 g for the Module A and B, respectively.

### Experimental Set-Up and Protocol

In order to investigate the performances of the two different modules, several tests were carried out by using an *ad hoc* experimental set-up. Four different characteristics have been experimentally evaluated and compared with the performances achieved by the original STIFF-FLOP module: (i) variable stiffness at rest position; (ii) variable stiffness in bent configuration; (iii) workspace; (iv) shape locking capability.

The experimental set-up counts on parts that are used to drive the modules in all the tests (i.e., vacuum pump and air compressor) while specific equipment is introduced to perform the single tests (e.g., load cell, magnetic tracking system).

For driving the modules, a simple On/Off vacuum control was selected and implemented by using a vacuum pump (Oil Lubricated Rotary Vane Pumps MM56p2, D.V.P Vacuum Technology s.r.l., Carpanelli S.p.A.). The vacuum working state, measured by an absolute pressure sensor (SWCN-V01-P3-2, Camozzi Group), corresponds to 0.1 bar pressure level whereas the ambient pressure state is set to the atmospheric pressure (1.01325 bar). Regarding the fluidic actuation, the pressure inside each pair of chambers is controlled by a proportional pressure regulator directly connected to an air compressor (S.A. 30/6 type, Werther International Inc., Houston, USA). The specific tests are detailed in the subsections reported below.

#### Variable Stiffness at Rest Position

**Figure 5:**
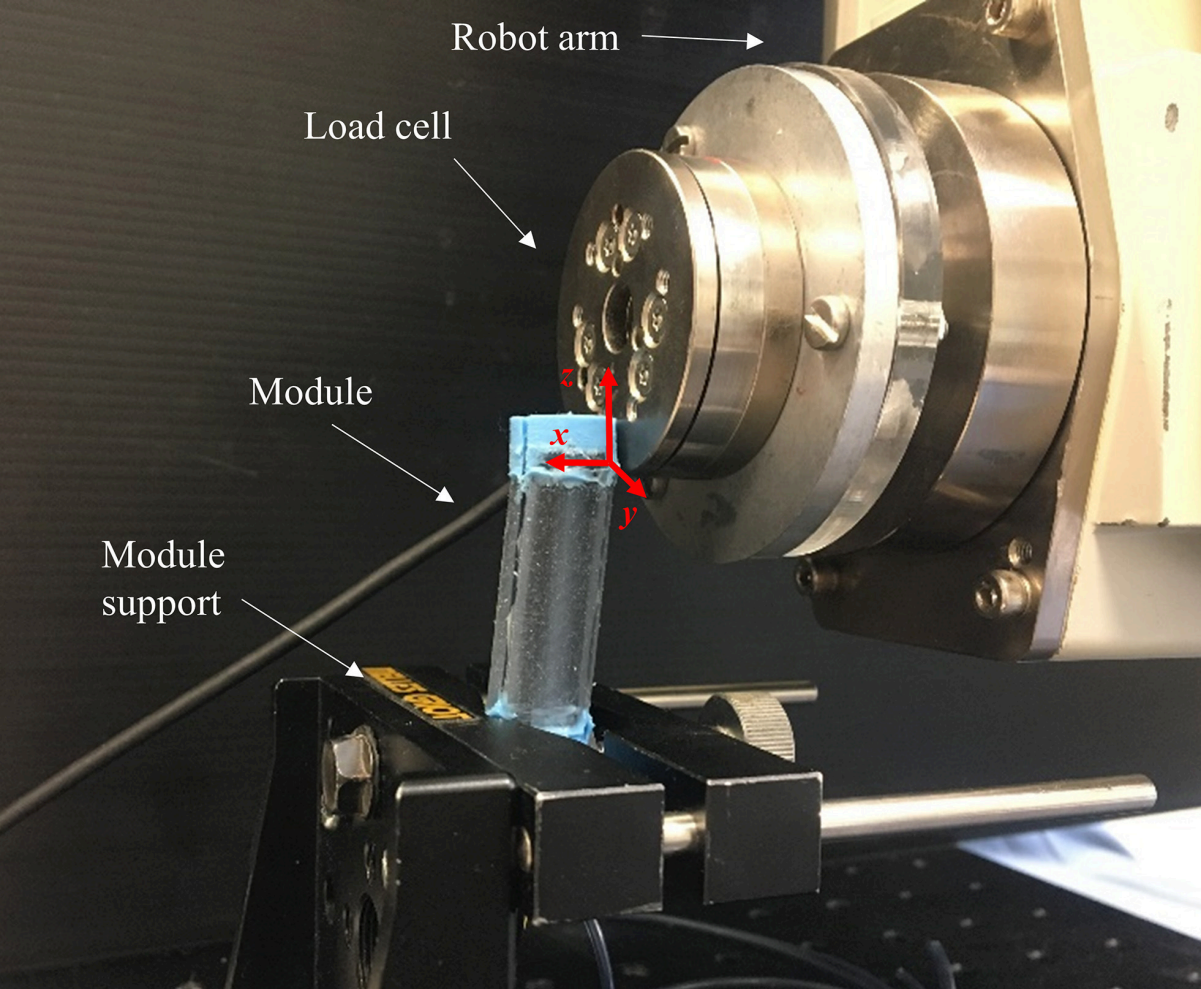
Setup for testing variable stiffness at rest position (Module A is reported as example).

This test needed an *ad-hoc* metallic housing to host the base of the two modules (i.e., Module A and Module B). The module tip was deflected horizontally (along the x-axis referring to the reference Cartesian coordinate system of Figure 5) by an anthropomorphic robotic arm with six DoFs (RV-6SL; Mitsubishi Electric) for a distance of 15 mm at a speed of 5 mm/s, as shown in Figure 5. An ATI-mini 45 Force/Torque sensor (ATI Industrial Automation, USA), mounted on the end-effector of the robotic arm, measured the resistive force developed by each module. The interaction surface between the module tip and the force sensor is properly defined for each test such that the application point of the force by the load cell to the module is kept as the origin of the reference Cartesian coordinate system, guaranteeing the same working conditions for all the experimental sessions. This point was conventionally defined as the lowest edge of the ATI-mini 45 sensor in contact with the most rigid silicone part of the module (the blue one). This point represents the origin of the reference Cartesian coordinate system and it is horizontally centered and vertically positioned 5 mm below the module tip. In order to guarantee the same setup for all the bending tests, the application point is manually reached before each test session. This experimental setup was controlled with a LabVIEW GUI (LabVIEW System Design Software—National Instrument), also used for data recording (sample rate of 10 kHz). Ten experimental trials were performed for each module: five tests keeping the stiffening chamber at atmospheric pressure (i.e., 1.01325 bar) and five under vacuum conditions (i.e., 0.1 bar). These tests are carried out for quantifying the contribution of the fiber jamming transition to the overall stiffness of the modules when at rest position.

#### Variable Stiffness in Bent Configuration

**Figure 6:**
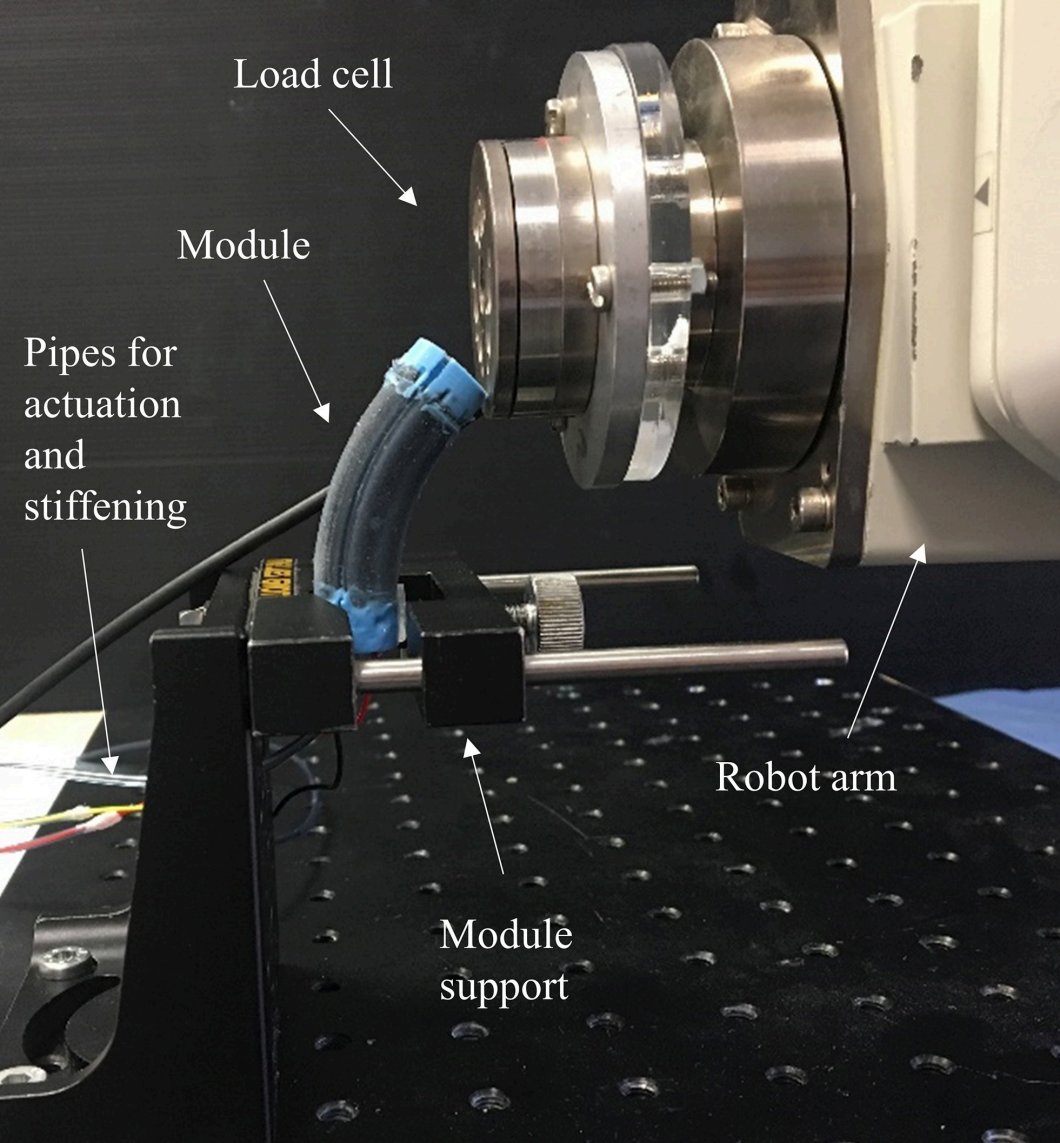
Setup for testing variable stiffness in bent configuration (Module A is reported as example).

The same setup has been used to quantify variable stiffness in bent configuration (Figure 6), but a different protocol has been followed. Before applying the lateral load, one MP of each module is activated using 1 bar pressure. The robot arm, equipped with the load cell, pushes the module for 10 mm at 5 mm/s velocity. Five trials have been carried out with the jamming system at atmospheric pressure, and five with the application of vacuum conditions. The application point is the same of the test mentioned above.

Maximum force for all tests are recorded and compared; the stiffness variation is also related to the previously tested configuration in order to evaluate if the deformed state of the module affects the stiffening capability of the fiber jamming system.

#### Module's Workspace

**Figure 7:**
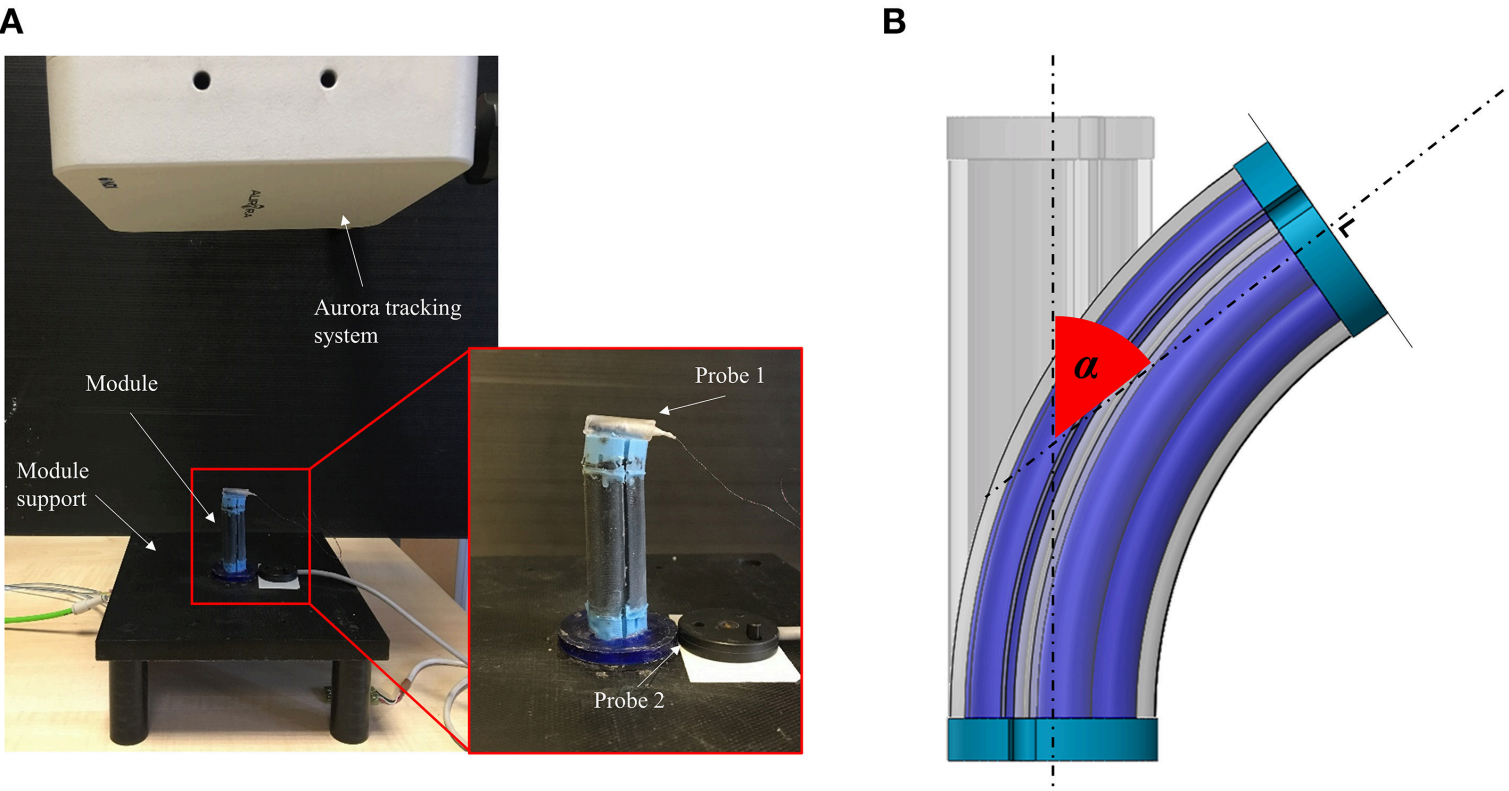
**(A)** Test setup used for workspace and shape locking evaluation (Module A is reported as an example) and **(B)** bending angle (α) evaluation method.

To evaluate the module's workspace, the bending angle has been measured in relation with the applied pressure. The input air pressure ranges from 0 to 1.2 bar with an increment of 0.2 bar and inflates a pair of chambers for each module. The workspace is evaluated as the capability of each chamber to bend the module on a single plane. An electro-magnetic system (NDI Medical Aurora Northern Digital Inc., Waterloo, Canada), with 0.48 mm as maximum accuracy, was used as a ground truth pose measuring device. In particular, one Aurora Mini 6 DOF Sensor (1.8 × 9 mm) was fixed on the tip of the module while an Aurora 6 DOF Reference probe (25 mm Disc) is located on the module support, close to the module base (Figure 7A). The two probes are used to monitor the position and orientation of the module tip with respect to the base. By using the ground truth system, the bending angle (α) is derived as the angle between the vectors normal to the module base and tip surfaces on the bending plane (Figure 7B).

A total of five trials were conducted for each module activating only a pair of chambers. This test aims at evaluating how much the presence of the fibers affects the dexterity of the manipulator.

#### Shape Locking

The same setup described for the workspace evaluation has been used for the shape locking tests following a multiphase procedure:

- The module is bent supplying a 1.2 bar pressure to a pair of chambers
- The angle α _1_ is recorded
- The vacuum is applied to the stiffening chamber for 30 s
- The pressure is removed from the fluidic chambers
- The angle α _2_ is recorded.

Five tests for Modules A and B have been carried out.

## Results and Discussion

In this section, the results obtained for each experimental test are reported and discussed. The analysis is based on the comparison between the performances of the two proposed designs with respect to the original STIFF-FLOP module.

### Variable Stiffness at Rest Position

Table 2 summarizes the results concerning the maximum stiffness variation that can be achieved for each configuration. For a comprehensive comparative analysis of the system performances, the maximum force developed by the original STIFF-FLOP module is reported as reference.

**Table 2:** Results of the bending tests carried out at rest configuration to evaluate the maximum stiffness variation for the three designs [i.e., original STIFF-FLOP module (Abidi et al., 2018), Module A and Module B].

**Configuration**	**Maximum force (Vacuum off) [N]**	**Maximum force (Vacuum on) [N]**	**Stiffness variation**
STIFF-FLOP module	0.36 ± 0.03	-	-
			
Module A	0.47 ± 0.02	0.57 ± 0.04	22 ± 15%
			
Module B	0.33 ± 0.02	0.85 ± 0.03	160 ± 28%

A first observation concerns the maximum force generated by each single module when the vacuum is not applied. It is reasonable that the original STIFF-FLOP module presents a lower maximum force with respect to Module A, because of the introduction of fibers in the inner free lumen. This change in the design can introduce an additional resistance to the bending motion that explains a value of 0.47 N for Module A with respect to 0.36 N of the original STIFF-FLOP module. Furthermore, the maximum force of the Module B is less than the maximum force of the other two configurations because the Module B has only one MP (i.e., two actuation chambers) instead of three (i.e., six actuation chambers) and two actuation chambers are more rigid than a chamber filled with fibers. This may seem counterintuitive, but, referring to Figure 2, it is easy to see that the cross section of Module A relies on a larger part of silicone that opposes to tensile forces developed during bending. In Module B, the most of the silicone is substituted by flexible fibers that can easily slide and bend when free to move.

The last column of the Table 2 reports the ratio between the maximum force measured in the jammed and the unjammed condition. This is the most appropriate parameter to assess the module performances and to provide a direct comparison of stiffening capability. The stiffening performance has a trend that is coherent with the design of the modules, namely the stiffness variation increases with the volume of the stiffening chamber and the number of fibers. Module A contains 8 fibers and presents a very limited stiffness variation with respect to Module B, which contains 14 fibers for each stiffening chamber (28 in total). Moreover, the location of the fibers plays a significant role. The bending moment of inertia of the module increases much more if the fibers are placed in the outer part of the cross section rather than in the central part.

In addition to these quantitative data, it is worth reporting that during these tests, once the imposed displacement is completely removed and no vacuum is applied, the modules equipped with fibers completely recover their initial position without showing any permanent deformations. This means that fibers remain free to move also when the module is deformed if there is no fluidic input.

### Variable Stiffness in Bent Configuration

The dominant role played by the fiber jamming is also confirmed in the stiffening test in bent configuration. Module B shows a remarkable stiffness variation, while Module A seems to be barely affected by the activation of the fiber jamming system, as reported in Table 3. The presence of two stiffening chambers enhances the module ability to keep its shape against external disturbances.

**Table 3:** Stiffness variation obtained in bent configuration for the two modules.

**Configuration**	**Maximum force (Vacuum off) [N]**	**Maximum force (Vacuum on) [N]**	**Stiffness variation**
Module A	0.28 ± 0.03	0.29 ± 0.02	Statistically irrelevant
			
Module B	0.36 ± 0.04	0.70 ± 0.03	99 ± 29%

These data, together with those reported in section Variable stiffness at rest position, support the overall concept of using such kind of modules to provide stability to the distal segment of a two-module soft manipulator. The substantial stiffness variation of the proximal module demonstrates the ability to compensate external disturbances providing stability to the distal module that instead remains more dexterous and flexible.

### Workspace

**Figure 8:**
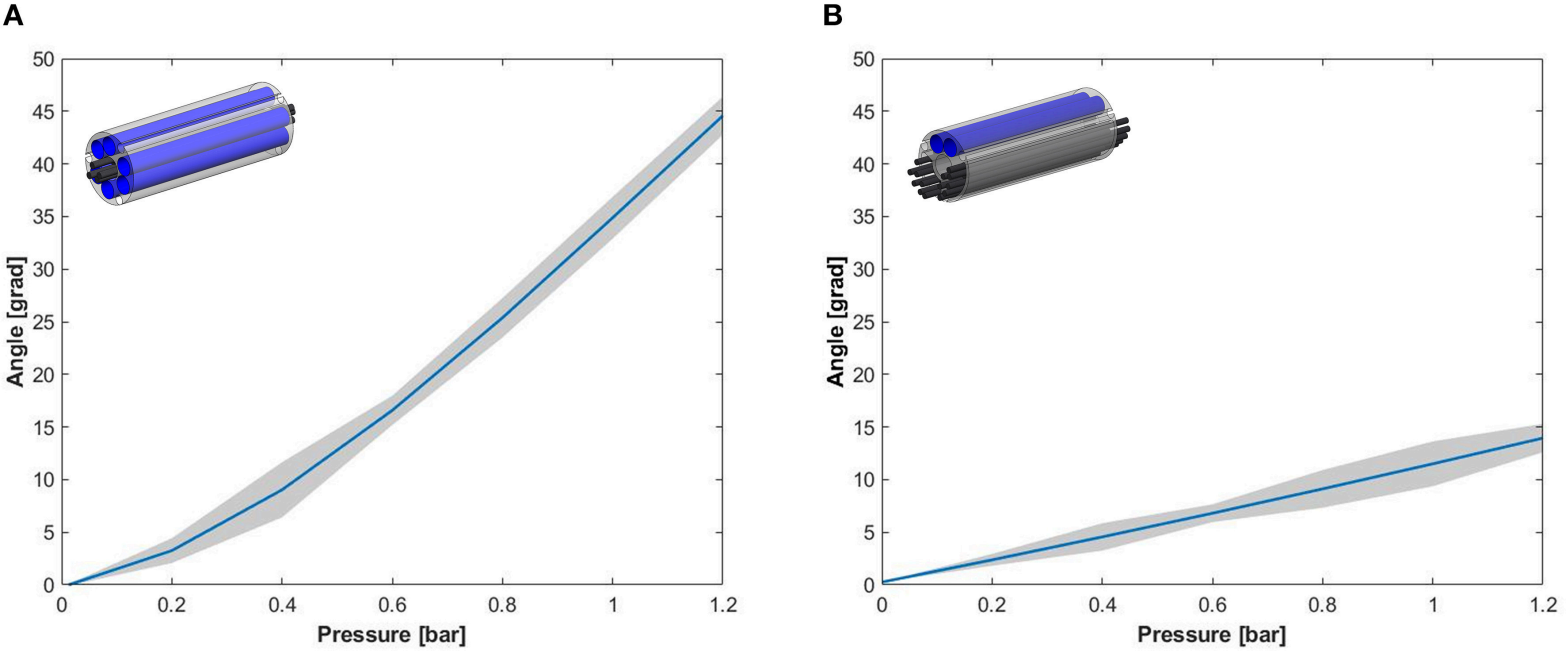
Experimental results for the angular workspace obtained through the activation of a single pair of chambers of **(A)** Module A and **(B)** Module B.

The results related to the workspace are limited to the evaluation of the module performances on a single bending plane. In particular, Figure 8 shows the angles achieved by the module tip at increasing pressure. For a given pressure, the bending angle reached by Module B is considerably lower with respect to the Module A. This different response might be due to the structural role played by the fibers in the module. In both the cases the fiber jamming system is not active, but the fibers experience a sliding motion that is subject to friction. However, Module B includes many more fibers and they are arranged in a way that the area moment of inertia is much higher, thus causing a stiffer structure.

Observing the performances in terms of workspace for both modules and comparing the results with the STIFF-FLOP module (i.e., the bending angle is 132.2° at 1.2 bar Abidi et al., 2018), the integration of the fibers considerably decreases the module workspace. This effect was predictable and is supported by the data about stiffness variation, but this is an acceptable limitation for the intended application. In this work, the main aim is to improve the effectiveness of the two-module surgical manipulator by increasing its stability and this can be done by using a proximal module equipped with the stiffening systems (sacrificing part of the workspace) and a distal one with high dexterity and no stiffening capabilities.

### Shape Locking Capability

The shape locking capability has been evaluated as the residual bending angle the module is able to maintain once the vacuum in the stiffness chambers is activated and the pressure in the fluidic chambers is removed. This effect is strictly related to the amount of fibers that are involved in the jamming transition phenomenon.

As expected, Module B presents a higher residual bending angle (Table 4). This module relies on a higher number of fibers and, looking at its overall design, its body has less silicone parts that in general give a large contribution in the elastic return of the module (i.e., recovery of its initial configuration once the deformation has been removed).

**Table 4:** The table summarizes the residual angles measured for the two modules.

**Configuration**	**α_1_ Angle @ 1.2 bar & Vacuum off**	**α _**2**_ Angle @ 0 bar & Vacuum on**	**Residual angle $\;\frac{\;\alpha \;2}{\;\alpha \;1}\;\times 100$**
Module A	46.66 ± 1.21°	9.31 ± 0.74°	20 ± 2.1%
			
Module B	14.80 ± 1.06°	10.32 ± 0.80°	70.18 ± 10.7%

### Overall Comparison

**Figure 9:**
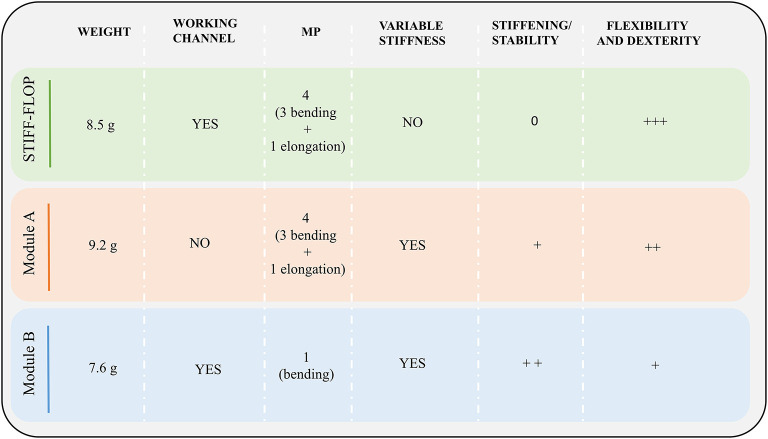
Module features: for each module architecture, weight, the presence of the inner free lumen (i.e., working channel), the number of MPs, the integration of a variable stiffness mechanism and the performance in terms of stability and flexibility are reported.

The results of the comparative analysis are summarized in Figure 9. Considering the STIFF-FLOP module as a reference starting point, both modules represent an improvement as they both demonstrated stiffening capability. However, this came with a significant reduction on flexibility and dexterity. This affected Module B more than Module A, mainly because of the higher number of fibers that have been integrated. It implies that the stiffening/stability is better for Module B (being directly proportional to the number of fibers), but to the detriment of dexterity. The only exception is represented by the passive tests (reported in Table 2) where the presence of the fibers seems to have a negative effect more on Module A than B. This suggests that: the presence of the fiber jamming system tends to make the module more flexible, but if a fluidic input is applied (whether it be vacuum to the jamming-based system or inflation of the fluidic chambers), a stiffness variation is induced and this variation is directly proportional to the volume of the fiber jamming chamber. This means that the fiber jamming system influences the behavior of the module both through direct or indirect activation.

Having a look at the system in terms of MPs and operational functionalities that a surgical manipulator should have in order to augment surgeon's abilities, Module A does not alter the motion capabilities of the STIFF-FLOP module, the central free lumen can no more be used for instrument insertion. Module B keeps the internal free lumen, but can only bend in one direction, meaning that the rotation of the supporting rod (the roll degree of freedom) must be enabled to restore omnidirectional bending (with severe implications on maneuverability).

The results highlight that, so far, there is no optimal solution that satisfies all the desired requirements in terms of miniaturized dimensions, free lumen for passing tools up to the tip, stiffness variation, flexibility and dexterity. The approach used in this work revealed an inverse relation between stiffness variation and motion performances, thus an optimal balance should be identified on the base of the target application. In particular, stiffness, and motion capabilities can be tuned in order to guarantee dexterity and flexibility for a soft and delicate navigation within the human body until the target district for the surgical task (e.g., retraction, suturing, and dissection) is reached, where on contrary stiffening is required for an effective force transmission.

## Conclusion and Future Work

Starting from the preliminary results obtained by Brancadoro et al. (2018), here we presented a possible exploitation of the fiber jamming transition technology as a variable stiffness mechanism integrated in the STIFF-FLOP soft manipulator. The STIFF-FLOP original module has been re-designed following two different approaches. The two new modules have been evaluated in terms of dexterity and variable stiffness capability. A comparative analysis has been carried out to study to what extent these two characteristics influence each other and to identify suitable compromises.

Results allowed defining the layout that presents the better trade-off between technical requirements and stiffness variation. A further outcome of the present study regards the awareness that the technology still needs further studies to be mastered, to define protocols and to standardize the manufacturing procedure, which so far is carried out through multiple manual steps as summarized in Figure 4.

In this view, the fiber jamming technology demonstrated to have suitable features for enabling a stiffness variation in soft bodied devices and in our specific case it is facilitating the shift from an endoscopic tool (mainly devoted to inspection and whose main requirements are dexterity, maneuverability and safe interaction), to a surgical tool (that should be able to transfer effective forces to the tissues and stabilization).

While the performances of the original STIFF-FLOP soft manipulator used as an endoscopic tool have been already proved in cadaver tests, the new capabilities enabled by the introduction of the fiber jamming technology still need to be tested in a real environment. Future activities will be focused on the assessment of the effectiveness and the advantages of such an approach in real surgical procedures (such as organ retraction, suturing, or dissection). On the other side, further studies on the physical principle at the base of fiber jamming transition itself could elucidate the main parameters affecting its performances and help defining design guidelines for the use of this technology.

## Author Contributions

MB, MM, and FG designed and fabricated the modules. All authors designed the experiments. MB and FG implemented the scenario and carried out the tests and the data analysis is made by MB, FG, and ST. MB, MM, and MC wrote the manuscript. ST, AM, and MC supervised the study.

### Conflict of Interest Statement

The authors declare that the research was conducted in the absence of any commercial or financial relationships that could be construed as a potential conflict of interest.

## References

[B1] Abidi H. Gerboni G. Brancadoro M. Fras J. Diodato A. Cianchetti M.. (2018). Highly dexterous 2-module soft robot for intra-organ navigation in minimally invasive surgery. Int. J. Med. Robot. Comput. Assist. Surg. 14:e1875. 10.1002/rcs.1875 29205769

[B2] Amend J. Cheng N. Fakhouri S. Culley B. (2016). Soft robotics commercialization: jamming grippers from research to product. Soft. Robot. 3, 213–222. 10.1089/soro.2016.0021 28078197 PMC5180083

[B3] Arezzo A. Mintz Y. Allaix M. E. Arolfo S. Bonino M. Gerboni G.. (2017). Total mesorectal excision using a soft and flexible robotic arm: a feasibility study in cadaver models. Surg. Endosc. 31, 264–273. 10.1007/s00464-016-4967-x 27338578

[B4] Behringer R. P. Chakraborty B. (2018). The Physics of Jamming for Granular Materials: A Review. Reports on Progress in Physics. 30132446 10.1088/1361-6633/aadc3c

[B5] Brancadoro M. Manti M. Tognarelli S. Cianchetti M. (2018). Preliminary experimental study on variable stiffness structures based on fiber jamming for soft robots, in 2018 IEEE International Conference on Soft Robotics (RoboSoft) (Livorno: IEEE).

[B6] Diodato A. Brancadoro M. De Rossi G. Abidi H. Dall'Alba D. Muradore R.. (2018). Soft robotic manipulator for improving dexterity in minimally invasive surgery. Surg. Innov. 25, 69–76. 10.1177/1553350617745953 29303068

[B7] Follmer S. Leithinger D. Olwal A. Cheng N. Ishii H. (2012). Jamming user interfaces: programmable particle stiffness and sensing for malleable and shape-changing devices, in Proceedings of the 25th Annual ACM Symposium on User Interface Software and Technology (Massachusetts, MA: ACM).

[B8] Fraś J. Czarnowski J. Maciaś M. Główka J. Cianchetti M. Menciassi A. (2015). New STIFF-FLOP module construction idea for improved actuation and sensing, in 2015 IEEE International Conference on Robotics and Automation (ICRA) (Seattle, WA:IEEE), 2901–2906. 10.1109/ICRA.2015.7139595

[B9] Kaufhold T. Böhm V. Zimmermann K. (2012). Design of a miniaturized locomotion system with variable mechanical compliance based on amoeboid movement, in 2012 4th IEEE RAS and EMBS International Conference on Biomedical Robotics and Biomechatronics (BioRob) (Roma: IEEE), 1060–1065. 10.1109/BioRob.2012.6290779

[B10] Kim Y.-J. Cheng S. Kim S. Iagnemma K. (2013). A novel layer jamming mechanism with tunable stiffness capability for minimally invasive surgery. IEEE Trans. Robot. 29, 1031–1042. 10.1109/TRO.2013.2256313

[B11] Laschi C. Mazzolai B. (2016). Lessons from animals and plants: the symbiosis of morphological computation and soft robotics. IEEE Robot. Autom. Mag. 23, 107–114. 10.1109/MRA.2016.2582726

[B12] Laschi C. Mazzolai B. Cianchetti M. (2016). Soft robotics: technologies and systems pushing the boundaries of robot abilities. Sci. Robot. 1:eaah3690 10.1126/scirobotics.aah3690 33157856

[B13] Li M. Ranzani T. Sareh S. Seneviratne L. D. Dasgupta P. Wurdemann H. A. (2014). Multi-fingered haptic palpation utilizing granular jamming stiffness feedback actuators. Smart Mater. Struct. 23:095007 10.1088/0964-1726/23/9/095007

[B14] Liu A. J. Nagel S. R. (1998). Nonlinear dynamics: jamming is not just cool any more. Nature 396:21 10.1038/23819

[B15] Manti M. Cacucciolo V. Cianchetti M. (2016). Stiffening in soft robotics: a review of the state of the art. IEEE Robot. Autom. Mag. 23, 93–106. 10.1109/MRA.2016.2582718

[B16] Narang Y. S. Vlassak J. J. Howe R. D. (2018). Mechanically versatile soft machines through laminar jamming. Adv. Funct. Mater. 28:1707136 10.1002/adfm.201707136

[B17] Ou J. Yao L. Tauber D. Steimle J. Niiyama R. Ishii H. (2014). JamSheets: thin interfaces with tunable stiffness enabled by layer jamming, in Proceedings of the 8th International Conference on Tangible, Embedded and Embodied Interaction (Munich: ACM), 65–72.

[B18] Pfeifer R. Bongard J. (2006). How the Body Shapes the Way We Think: A New View of Intelligence. Kadaikala: MIT Press.

[B19] Ranzani T. Gerboni G. Cianchetti M. Menciassi A. (2015). A bioinspired soft manipulator for minimally invasive surgery. Bioinspir. Biomimetics 10:035008. 10.1088/1748-3190/10/3/035008 25970550

[B20] Rus D. Tolley M. T. (2015). Design, fabrication and control of soft robots. Nature 521:467. 10.1038/nature14543 26017446

[B21] Shen H. (2016). Meet the soft, cuddly robots of the future. Nature 530:24. 10.1038/530024a 26842040

[B22] Stanley A. A. Gwilliam J. C. Okamura A. M. (2013). Haptic jamming: a deformable geometry, variable stiffness tactile display using pneumatics and particle jamming, in 2013 World Haptics Conference (WHC) (Daejeon: IEEE), 25–30. 10.1109/WHC.2013.6548379

[B23] Steltz E. Mozeika A. Rodenberg N. Brown E. Jaeger H. M. (2009). Jsel: Jamming skin enabled locomotion, in IEEE/RSJ International Conference on Intelligent Robots and Systems, 2009. IROS 2009 (St. Louis, MO: IEEE), 5672–5677. 10.1109/IROS.2009.5354790

[B24] Sun Y. Yap H. K. Liang X. Guo J. Qi P. Ang M. H. Jr. (2017). Stiffness customization and patterning for property modulation of silicone-based soft pneumatic actuators. Soft Robot. 4, 251–260. 10.1089/soro.2016.0047 29182082

[B25] Wall V. Deimel R. Brock O. (2015). Selective stiffening of soft actuators based on jamming, in 2015 IEEE International Conference on Robotics and Automation (ICRA) (Seattle, WA: IEEE), 252–257. 10.1109/ICRA.2015.7139008

[B26] Wang L. Yang Y. Chen Y. Majidi C. Iida F. Askounis E. (2018). Controllable and reversible tuning of material rigidity for robot applications. Materials Today 21, 563–576. 10.1016/j.mattod.2017.10.010

[B27] Zambrano D. Cianchetti M. Laschi C. Hauser H. Füchslin R. Pfeifer R. (2014). The morphological computation principles as a new paradigm for robotic design. Opin. Outlooks Morphol. Comput. 214–225. 10.13140/2.1.1059.4242

